# Dissecting the Role of SAL1 in Metabolizing the Stress Signaling Molecule 3′-Phosphoadenosine 5′-Phosphate in Different Cell Compartments

**DOI:** 10.3389/fmolb.2021.763795

**Published:** 2022-01-21

**Authors:** Natallia Ashykhmina, Kai Xun Chan, Henning Frerigmann, Frank Van Breusegem, Stanislav Kopriva, Ulf-Ingo Flügge, Tamara Gigolashvili

**Affiliations:** ^1^ Institute for Plant Sciences, Cologne Biocenter, University of Cologne, Cologne, Germany; ^2^ Department of Plant Biotechnology and Bioinformatics, Ghent University, Ghent, Belgium; ^3^ VIB Center for Plant Systems Biology, Ghent, Belgium; ^4^ Max Planck Institute for Plant Breeding Research, Cologne, Germany

**Keywords:** 3′-phosphoadenosine 5′-phosphate (PAP), chloroplasts, mitochondria, nuclei, cytosol, metabolite signaling, SAL1, nucleotidase/phosphatase

## Abstract

Plants possess the most highly compartmentalized eukaryotic cells. To coordinate their intracellular functions, plastids and the mitochondria are dependent on the flow of information to and from the nuclei, known as retrograde and anterograde signals. One mobile retrograde signaling molecule is the monophosphate 3′-phosphoadenosine 5′-phosphate (PAP), which is mainly produced from 3′-phosphoadenosine 5′-phosphosulfate (PAPS) in the cytosol and regulates the expression of a set of nuclear genes that modulate plant growth in response to biotic and abiotic stresses. The adenosine bisphosphate phosphatase enzyme SAL1 dephosphorylates PAP to AMP in plastids and the mitochondria, but can also rescue *sal1 Arabidopsis* phenotypes (PAP accumulation, leaf morphology, growth, etc.) when expressed in the cytosol and the nucleus. To understand better the roles of the SAL1 protein in chloroplasts, the mitochondria, nuclei, and the cytosol, we have attempted to complement the *sal1* mutant by specifically cargoing the transgenic SAL1 protein to these four cell compartments. Overexpression of SAL1 protein targeted to the nucleus or the mitochondria alone, or co-targeted to chloroplasts and the mitochondria, complemented most aspects of the *sal1* phenotypes. Notably, targeting SAL1 to chloroplasts or the cytosol did not effectively rescue the *sal1* phenotypes as these transgenic lines accumulated very low levels of SAL1 protein despite overexpressing *SAL1* mRNA, suggesting a possibly lower stability of the SAL1 protein in these compartments. The diverse transgenic *SAL1* lines exhibited a range of PAP levels. The latter needs to reach certain thresholds in the cell for its impacts on different processes such as leaf growth, regulation of rosette morphology, sulfate homeostasis, and glucosinolate biosynthesis. Collectively, these findings provide an initial platform for further dissection of the role of the SAL1–PAP pathway in different cellular processes under stress conditions.

## 1 Introduction

Eukaryotic cells are highly organized into different compartments, such as the mitochondria, the endoplasmic reticulum, peroxisomes, and the Golgi apparatus. Additionally, plant cells possess plastids, large vacuoles, and the apoplast, each with a unique set of enzymes and functions. Chloroplasts not only perform photosynthesis but also participate in the assimilation of mineral nutrients (e.g., S, N, and P) and synthesize numerous compounds, including secondary metabolites [phenylpropanoids and glucosinolates (GSLs)], fatty acids, and amino acids (Bhardwaj et al., 2015). Mitochondria are essential for cellular respiration and contribute to the generation of reactive oxygen species (ROS) (Wang et al., 2018). The major protein complexes of chloroplasts and the mitochondria are combinations of nuclear- and organelle-encoded subunits; therefore, appropriate gene expression involves a tight coordination between the nucleus and organelles. Plastids and the mitochondria produce retrograde signals that modulate nuclear gene expression and organellar biogenesis or optimize their performance (Chi et al., 2015). Many signals in organellar retrograde pathways have been identified, including chlorophyll intermediates, ROS, and other metabolites (Chan et al., 2016; Ishiga et al., 2017; Pesaresi and Kim, 2019). For example, the SAL1–PAP retrograde signaling pathway is implicated in responses to drought and high-light stresses (Estavillo et al., 2011). The retrograde signaling molecule PAP (3′-phosphoadenosine 5′-phosphate) is generated by the sulfate assimilation pathway and degraded by SAL1 into AMP and inorganic phosphate. During stress conditions, PAP accumulates, as it can no longer be degraded by the nucleotidase/phosphatase SAL1, which becomes inactivated by oxidation (Chan et al., 2016). Transcriptome analysis has also shown that SAL1 regulates an overlapping set of genes with 3′ exoribonucleases (XRNs), suggesting that they function in a common signaling pathway (Estavillo et al., 2011).

The nuclear-encoded SAL1 belongs to a small family of six nucleotidase/phosphatase proteins in *Arabidopsis thaliana*, and out of these six proteins, only SAL1 contains a dual-targeting signal, which directs the protein to both the mitochondria and chloroplasts. Previous research has partly elucidated the role of SAL1 in sulfur metabolism and retrograde signaling (Lee et al., 2012; Chan et al., 2013). Several *sal1* mutant alleles have been identified by genetic screens: *fiery1* through an elevated abscisic acid (ABA) response (Xiong et al., 2001), *alx8* was identified in a screen for the elevated expression of *ASCORBATE PEROXIDASE2* at high- and low-light conditions, *fou8* possesses elevated jasmonic acid (JA) levels (Rodríguez et al., 2010), and *ron1* was isolated from a screen for mutants with aberrant vascular patterning (Robles et al., 2010) and has rounder leaves and altered auxin signaling. The *alx8* and *fry1* alleles can rescue stomatal closure in ABA-insensitive mutants, while *fou8* has a high jasmonate level (Rodríguez et al., 2010), suggesting that SAL1 regulates development and stress responses *via* at least three of the main phytohormone signaling pathways. Consistently, Ishiga et al. (2017) have recently shown that the SAL1–PAP pathway is important for the regulation of retrograde signaling in plant immunity and that the salicylic acid (SA) and JA pathways are compromised in *sal1*, thereby confirming a role for the SAL1–PAP pathway in the antagonistic interaction between SA and ABA, JA and ABA, and SA and JA.

**FIGURE 1:**
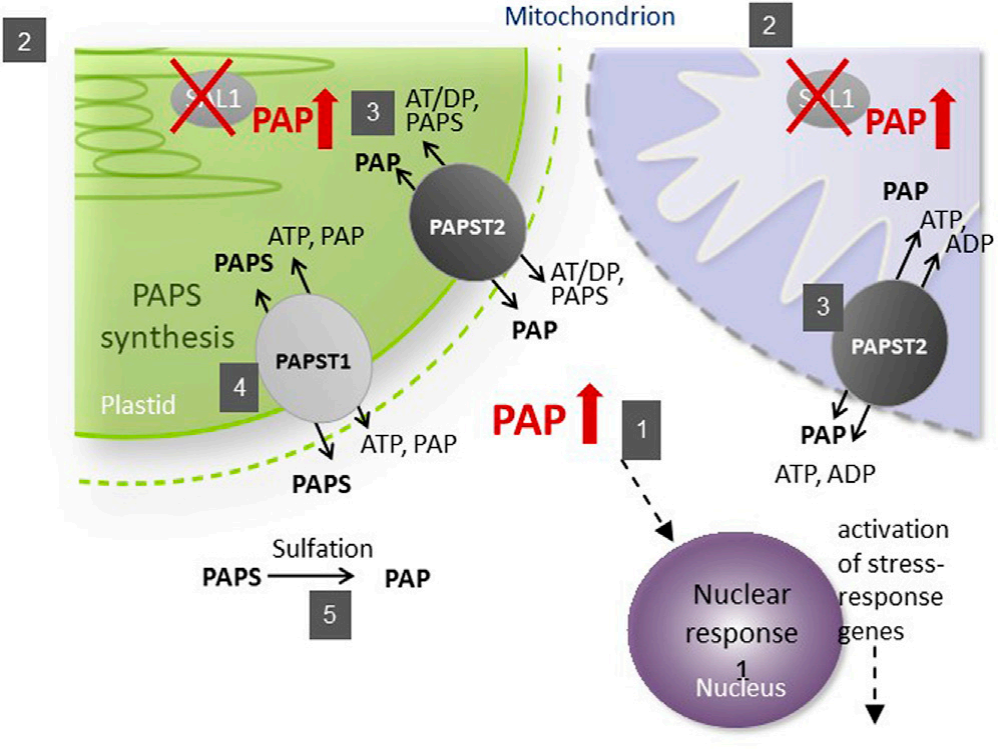
Schematic illustration of phosphoadenosine 5′-phosphate (PAP) catabolism in the *sal1* mutant. The SAL1 (*2*) protein is localized to plastids and the mitochondria, where it regulates the intercellular PAP concentration (*1*). PAPST2 (*3*) and PAPST1 (*4*) import cytosolic PAP into the mitochondria and plastids. PAPST1 (*4*) delivers newly synthesized PAPS from the plastid to the cytosol in exchange for PAP. In the cytosol and the Golgi apparatus, PAPS consumption *via* sulfotransferases results in PAP release (*5*). A defect in SAL1 activity leads to PAP accumulation in these organelles, which decelerates PAP uptake. The resulting increase in cytosolic PAP induces nuclear responses and phenotypes recorded previously for *sal1* mutants.

The subcellular localization of its components is important for the function of the SAL1–PAP pathway in retrograde signaling (Figure 1). As the precursor of PAP, 3′-phosphoadenosine 5′-phosphosulfate (PAPS) is mainly synthesized in chloroplasts and transported into the cytosol by PAPST1 (Gigolashvili et al., 2012) and, to a lesser extent, by PAPST2 (Tee, 2018; Ashykhmina et al., 2019), where it is used as a sulfate donor by sulfotransferases (SOTs) for various sulfation reactions. These reactions generate PAP in the cytosol, which is then transported into chloroplasts and the mitochondria for degradation by SAL1 (Estavillo et al., 2011). Unless it is transported back into organelles, PAP regulates nuclear gene expression (Dichtl et al., 1997) and inhibits SOTs in the cytosol (Rens-Domiano and Roth, 1987), thus modulating sulfur assimilation in plant cells (Lee et al., 2012; Chan et al., 2013). To modulate the concentration of PAP in the cytosol, its transport into chloroplasts and the mitochondria is mainly mediated by PAPST2 (Ashykhmina et al., 2019) and, to a lesser extent, by PAPST1 (Gigolashvili et al., 2012).

The dual localization of SAL1 in plastids and the mitochondria and the presence of two different PAP transporters in these compartments are indicative of distinct SAL1 functions in these two compartments. For example, studies that targeted yeast SAL1 (Sc-SAL1) to the chloroplast only (Rodríguez et al., 2010; Estavillo et al., 2011), as well as the nuclear- and cytosolic-specific expression of a truncated (missing chloroplastidic localization sequence) SAL1 construct (Kim and von Arnim 2009), complemented *sal1* mutant phenotypes to different extents. Furthermore, although the *papst1* mutant showed inhibited plant growth associated with lowered levels of PAPS and invariant levels of cellular PAP (Gigolashvili et al., 2012), the *papst2* transfer DNA (T-DNA) and artificial microRNA (amiRNA) mutant lines displayed a larger rosette than did the wild type and moderately increased PAP levels (Ashykhmina et al., 2019). Remarkably, morphological phenotypes in the high-PAP-accumulating *sal1* were partially restored to wild type in the *sal1 papst1* double mutant, coincident with a lower cytosolic PAP content and a higher chloroplastic PAP level than in *sal1*. This is due to a decrease in PAPS transport *via* PAPST1 and, consequently, a reduced PAP formation in the cytosol. In contrast, *sal1 papst2* showed an enhancement compared to the *sal1* phenotype, with higher cytosolic and chloroplastic PAP contents than *sal1*.

Collectively, the above observations suggest that PAP has complex effects on plant growth, not only depending on its dosage but also on the subcellular location. This intriguing hypothesis prompted us to deconvolute the role of SAL1 in different cell compartments by systematically expressing it in chloroplasts, the mitochondria, nuclei, and the cytosol and assessing its ability to complement the morphological, physiological, and chemical phenotypes of the *sal1* mutant (Ashykhmina et al., 2019).

## 2 Results

### 2.1 Generation of Chimeric Constructs to Direct *SAL1* Protein Into Different Cell Compartments

To achieve the compartment-specific accumulation of SAL1 and expression of *SAL1*, we generated six chimeric constructs (Supplementary Figure S1). Constructs *SAL1_I* to *SAL1_IV* consisted of a truncated *SAL1* (*SAL1tr*) backbone fused to various organellar targeting sequences. This *SAL1tr* backbone was previously reported to be localized to the cytosol and the nucleus and to complement the *sal1* phenotype, in the absence of organellar targets (Kim and von Arnim, 2009). *SAL1_I* was designed to express the protein exclusively in the nucleus and contained *SAL1tr* fused to a nuclear localization sequence (NLS) at the C-terminus. Construct *SAL1_II* was created to express *SAL1tr* exclusively in the cytosol and was a fusion of the *SAL1tr* backbone to the nucleus exclusion sequence (NES) from At1g07140. This NES was necessary to avoid *SAL1* localization to the nuclei, as described by Kim and von Arnim (2009). To constructs II–V, we added NES at the C-terminus of *SAL1* by incorporating it into primers by PCR. Construct *SAL1_III* was designed to express *SAL1* exclusively in plastids and consisted of *SAL1tr* fused to the chloroplast pre-sequence (cPS) of the Rubisco small subunit (SSU) at the N-terminus and to the NES at the C-terminus. Construct *SAL1_IV* aimed to express SAL1 exclusively in the mitochondria and contained *SAL1tr* fused to the mitochondrial pre-sequence (mPS) or transit peptide of heat shock protein 90 (Hsp90) (Krishna and Gloor, 2001) at the N-terminus and NES at the C-terminus. Construct *SAL1_V* encoded the native *SAL1* pre-sequence and full-length SAL1 fused to the NES at the C-terminus and should target *SAL1* to plastids and the mitochondria. *SAL1tr*, as described by Kim and von Arnim (2009), was designated *SAL_VI.*

**FIGURE 2:**
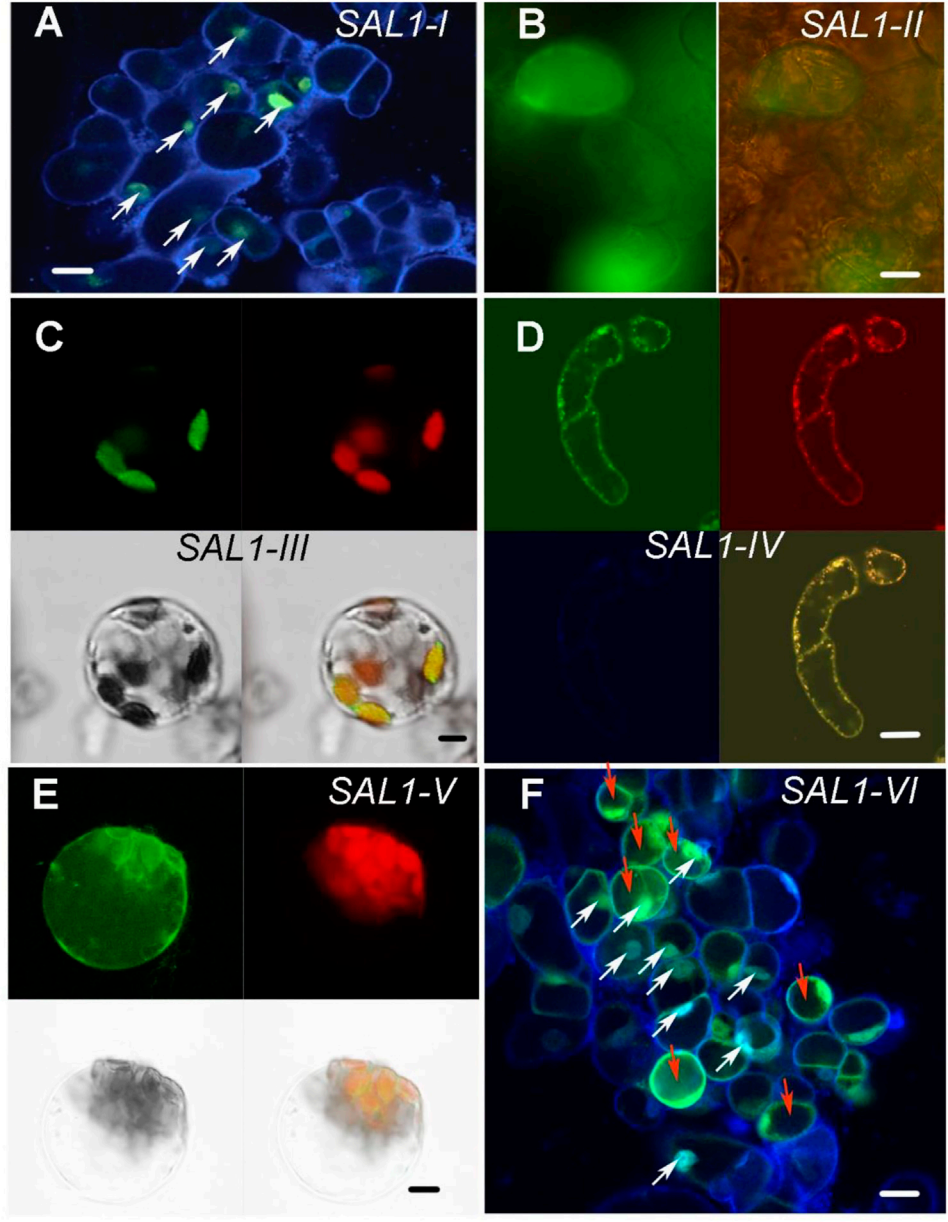
Subcellular localization of *SAL1_I*–*SAL1_VI* proteins in *Arabidopsis* root cell suspension cultures, protoplasts from the mesophyll, and in *Nicotiana benthamiana*. Transient expression of SAL1 protein chimeras fused to green fluorescent protein (GFP) under control of the *35S CaMV* promoter in various subcellular compartments of cells from *Arabidopsis* root cell suspension culture, as revealed by confocal fluorescence microscopy. **(A)** DAPI-stained *Arabidopsis* suspension cells form roots showing the nuclear localization of SAL1_I:GFP. *White arrows* show cells in which both GFP and DAPI are visible in the nuclei. *Bar* = 20 μm. **(B)** Cytosolic localization of SAL1_II:GFP shown with GFP filter and bright field and GFP. *Bar* = 40 μm. **(C)** Chloroplastic localization of SAL1_III:GFP in *Arabidopsis* protoplasts form the mesophyll four fields with GFP, chlorophyll autofluorescence, bright field, and overlay of GFP and chlorophyll signals. *Bar* = 10 μm. **(D)** Mitochondrial localization of SAL1_IV:GFP in *Arabidopsis* suspension cells form roots four fields with GFP, MitoTracer in *red*, DAPI staining, and overlay of both MitoTracker with GFP. *Bar* = 20 μm. **(E)** SAL1_V:GFP localization in *Arabidopsis* protoplasts form the mesophyll. It shows the presence of SAL1 in chloroplasts and in some tiny dot-like structures in the cytosol, which can be the mitochondria. Four fields show GFP, chlorophyll autofluorescence, bright field, and overlay of GFP and chlorophyll signals. *Bar* = 10 μm. **(F)** DAPI-stained *Arabidopsis* suspension cells form roots*,* showing both the cytosolic and nuclear localization of GFP. *White arrows* show cells in which both GFP and DAPI are visible in the nuclei. *Red arrows* show cells in which GFP is visible in the cytosol. *Bars* = 20 μm.

To confirm the subcellular localization of *SAL1* fusion proteins experimentally, we used *Arabidopsis* root cell suspension cultures and mesophyll protoplasts (Figure 2 and Supplementary Figure S2). Suspension cells from *Arabidopsis* roots were transformed with *Agrobacterium* carrying constructs encoding SAL1_I–VI fused to green fluorescent protein (GFP) at the C-terminus (Berger et al., 2007), and protoplasts were isolated from the mesophyll of leaves and transfected using purified plasmid DNA (Yoo et al., 2007). Fluorescence confocal microscopic analysis, shown in Figure 2 and Supplementary Figure S2, confirmed the expected localization of the transiently expressed SAL1_(I–VI):GFP fusion proteins in the designated compartments. DAPI staining of *Arabidopsis* root cells expressing *SAL1* *_I-GFP* and *SAL1_VI-GFP* (Figures 2A,F) confirmed the nuclear localization of both constructs (nuclei of cells containing both DAPI and *SAL1-GFP* are indicated by white arrows). The cytosolic localization of *SAL1_II-GFP* and *SAL1_VI-GFP* was observed in cells showing equal distribution of GFP in the cytosol (Figures 2B,F). Cells transfected with the *SAL1-VI* construct and showing SAL1-GFP in the cytosol are marked by red arrows (Figure 2F). The chloroplastidic localization of *SAL1_III_GFP* can be also confirmed (Figure 2C) as chlorophyll autofluorescence coincides with the GFP signal. Similarly, the mitochondrial localization of *SAL1_IV* (Figure 2D) was revealed by confirming the presence of the MitoTracker signal in the same structures as *SAL1_IV-GFP*. The GFP signal of *SAL1_V* was difficult to interpret as the expression level of this construct was weak (adjustments of levels were applied in Photoshop CS3). Nevertheless, the presence of *SAL1_V* protein in chloroplasts and in some mitochondria-like structures in the cytosol can be assumed (Figure 2E). The co-localization of *SAL1_V* with the MitoTracker was not successful as the GFP signal was too weak for this assay. Interestingly, the expression of *SAL1_V* in *Arabidopsis* suspension cells from roots, which lack chloroplasts (Supplementary Figure S2), showed intensive GFP staining in tiny mitochondria-like structures in the cytosol. In line with this observation Chen et al. (2011) and Estavillo et al. (2011) have previously demonstrated that when the full-length protein of SAL1 is fused to GFP (similar to the SAL1_V construct used in this work), SAL1 will be found in both chloroplasts and mitochondria. The only difference in the SAL1_V construct used in this work from that used by Chen et al. (2011) and Estavillo et al. (2011) is that our construct contained NES. However, as NES did not lead to mislocalization of constructs *SAL1_II*, *SAL1_III*, and *SAL1_IV*, the potential mislocalization of *SAL1_V-GFP* is less probable.

### 2.2 Selection of Partly and Fully Complemented Transgenic Mutant Lines With Varying *SAL1* Expression Levels

**FIGURE 3:**
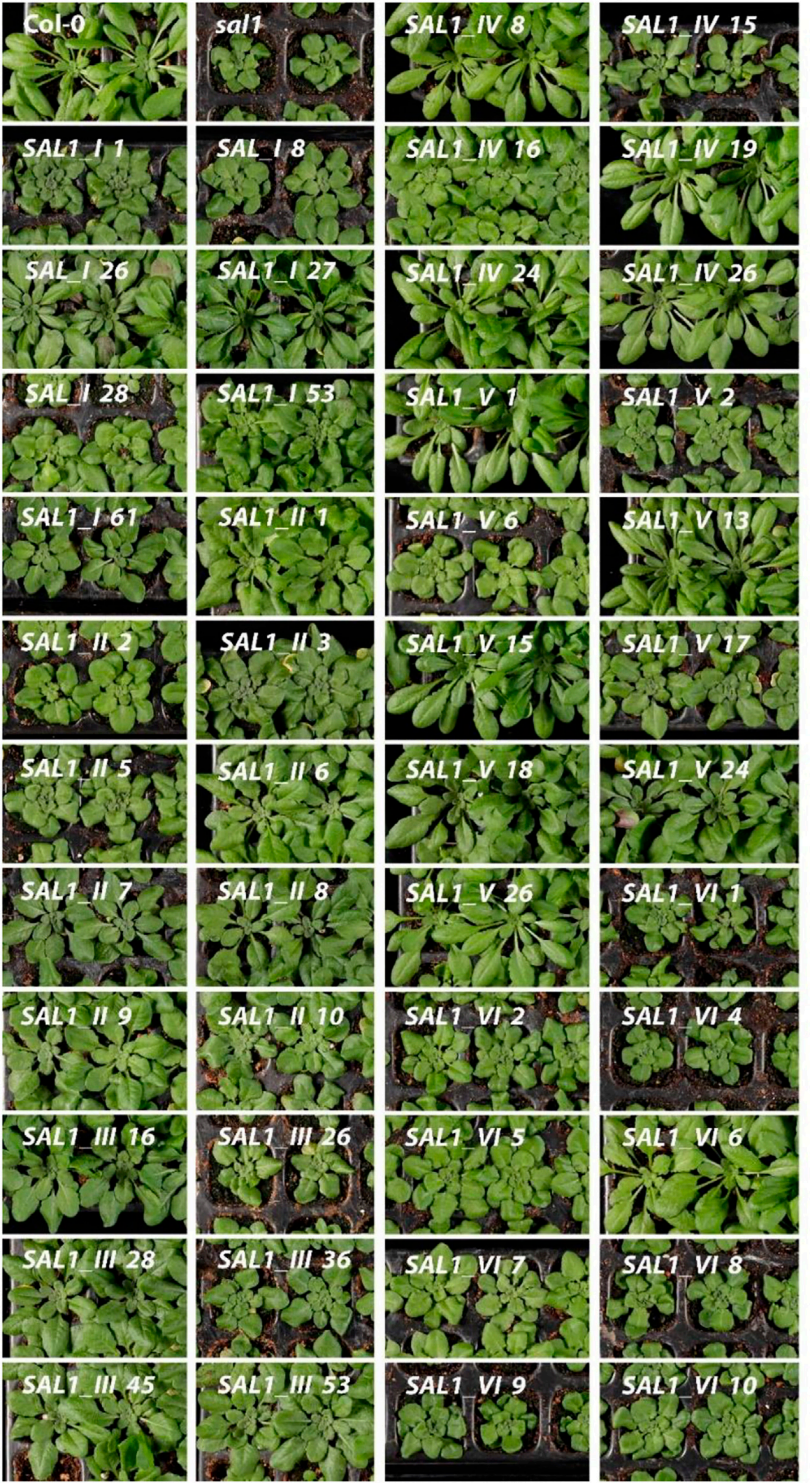
Morphological phenotypes of plants expressing *SAL1* chimeric constructs in different cell compartments. Plants were grown for 5 weeks on soil in short days in a controlled environment chamber. Bar = 2 cm. *SAL1_I*, nuclear localization; *SAL1_II*, cytosolic localization; *SAL1_III*, chloroplastic localization; *SAL1_IV*, mitochondrial localization; *SAL1_V*, chloroplastic and mitochondrial localization; and *SAL1_VI*, nuclear and cytosolic localization, which served as a positive control for the complementation (Kim and von Arnim, 2009).

**FIGURE 4:**
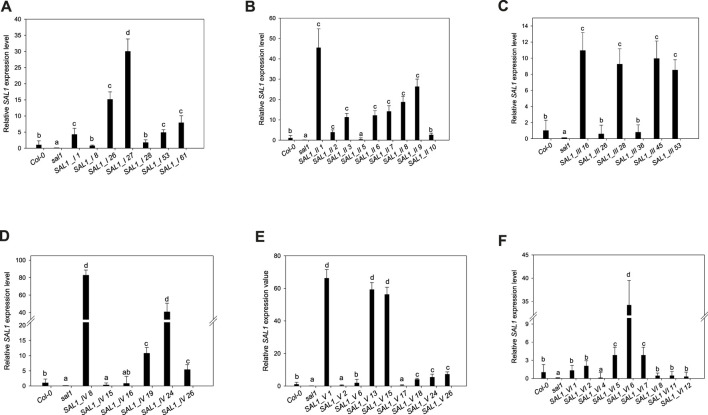
Expression levels of *SAL1* in different transgenic lines. Plants were grown for 5 weeks on soil in short days in a controlled environment chamber, mRNA was isolated, and the expression of *SAL1* analyzed. *SAL1* expression levels for *SAL1_I* (nuclear localization) **(A)**, *SAL1_II* (cytosolic localization) **(B)**, *SAL1_III* (chloroplastic localization) **(C)**, *SAL1_IV* (mitochondrial localization) **(D)**, **(E)** *SAL1_V* (chloroplastic and mitochondrial localization) **(E)**, and *SAL1_VI* (nuclear and cytosolic localization) **(F)**. These SAL1 expression data show the mean ± SE from two independent experiments with five biological replicates in each (*n* = 10). Relative expression values were normalized to *Actin2* and compared with the expression level in wild-type plants (Col-0 = 1). *Different letters* indicate significant differences among means based on *t*-tests at *p* < 0.05.

**FIGURE 5:**
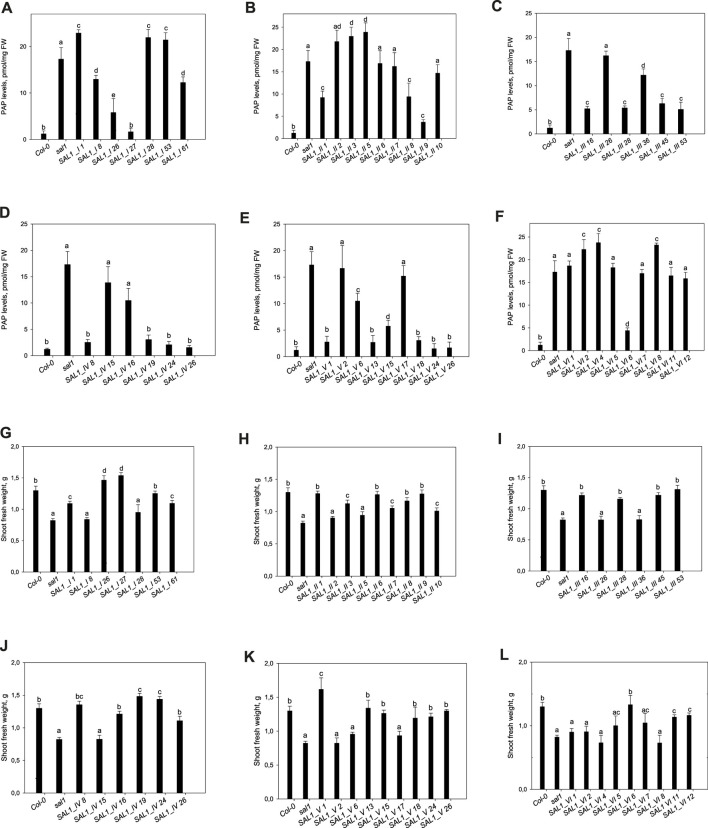
Phosphoadenosine 5′-phosphate (PAP) levels and shoot fresh weight in *sal1* mutants with the directed expression of *SAL1* to various cell compartments. **(A–F)** PAP levels in rosette leaves of 5-week-old *SAL1* and stably transformed *sal1* transgenic plants overexpressing SAL1 chimeric proteins. **(A)** *SAL1_I* (nuclear localization). **(B)** *SAL1_II* (cytosolic localization). **(C)** *SAL1_III* (chloroplastic localization). **(D)** *SAL1_IV* (mitochondrial localization). **(E)** *SAL1_V* (chloroplastic and mitochondrial localization). **(F)** *SAL1_VI* (nuclear and cytosolic localization). Plants were grown on soil in short days in a controlled environment chamber. Data show the mean ± SD (*n* = 3). *FW*, fresh weight. *Different letters* indicate significant differences among means based on *t*-tests at *p* < 0.05. **(G–L)** Shoot fresh weight of *sal1* mutants complemented with *SAL1* expressed in different cell compartments. Shoot fresh weight of wild-type, *sal1*, and stably transformed *sal1* transgenic plants overexpressing SAL1 chimeric proteins. **(G)** *SAL1_I* (nuclear localization). **(H)** *SAL1_II* (cytosolic localization). **(I)** *SAL1_III* (chloroplastic localization). **(J)** *SAL1_IV* (mitochondrial localization). **(K)** *SAL1_V* (chloroplastic and mitochondrial localization). **(L)** *SAL1_VI* (nuclear and cytosolic localization). Plants were grown for 4 weeks on MS agar plated under short-day conditions in an environment-controlled chamber. Data show the mean ± SD (*n* = 9). *Different letters* indicate significant differences among means based on *t*-tests at *p* < 0.05.

To study the function of *SAL1* in different cell compartments *in planta*, *sal1* mutant plants were transformed with the respective *SAL1* chimeric constructs. We selected independent transgenic lines that showed varying *SAL1* transcript levels and that were either marginally, moderately, or fully complemented in terms of the overall rosette morphology (Figure 3). We isolated 77 independent transgenic lines for each of *SAL1_I*, *SAL1_III*, and *SAL1_IV*, 40 lines for *SAL1_V*, and 12 lines for each of *SAL1_II* and *SAL1_VI*. Although we repeated the transformation process several times, we were not able to significantly increase the number of transgenic lines for constructs *SAL1_II* and *SAL1_VI*, indicating that SAL1 localization in the cytosol was probably not indiscriminate for the survival of transgenic plants. We then selected six to nine lines for each construct, which showed different levels of complementation. These lines were first compared to wild type and *sal1* in their visual appearance of rosette morphology, *SAL1* mRNA levels, shoot fresh weight (biomass) of fully developed 5-week-old adult plants, and PAP levels (Figures 3–5).

### 2.3 Complementation of PAP Levels and Biomass Following *SAL1* Expression in Different Compartments

#### 2.3.1 Nucleus

Seven independent lines with varying *SAL1* expression levels were analyzed in detail and are presented here. The extent to which the different *sal1* phenotypes were complemented correlated well with the degree of nuclear-targeted *SAL1* transcript levels. *SAL1_I* lines *26* and *27* showed the highest *SAL1* mRNA levels at 15- to 30-fold that of the wild type (Figure 4A). Adult plants of these lines were fully complemented in terms of rosette morphology, showed higher biomass than did the wild type, and had PAP levels that were either similar to those of the wild type or significantly decreased compared to those of *sal1* (Figures 4A, 5A,G). The *SAL1_I* lines *1*, *53*, and *61* with moderate *SAL1* overexpression (five to eight fold higher than that of the wild type) only showed partial complementation in rosette morphology, biomass, and PAP levels, whereas lines *8* and *28* with wild-type levels of *SAL1* expression only partially complemented leaf shape, but not biomass or PAP (Figures 4A, 5A,G).

#### 2.3.2 Cytosol

Following the localization of *SAL1* to the cytosol by construct *SAL1_II*, we obtained 12 independent transgenic lines with a stable phenotype. We analyzed nine lines that showed varying levels of *SAL1* expression in detail. All lines showed only partial complementation and a disconnect between their growth/development phenotypes and PAP levels, despite high the *SAL1* expression level in several transgenic lines (*SAL1_II 1*, *3*, *6*, *7*, *8*, and *9*) (Figures 4B, 5B,H). For instance, biomass was complemented to Col-0 levels in three out of nine lines (*SAL1_II 1*, *8*, and *9*) (Figure 5H), but both the PAP levels and morphological phenotype of these lines were intermediate between that of Col-0 and *sal1* (Figures 4B, 5B). Similarly, *SAL1_II* lines *3* and *7* showed partially complemented biomass (Figure 5H) despite accumulating similar or higher levels of PAP compared to *sal1* (Figure 5B). *SAL1_II* lines *2*, *3*, and *5* accumulated higher levels of PAP than did *sal1* and largely failed to restore biomass and rosette morphology (Figures 4B, 5H)*.* This observation was similar to that for the *SAL1-I* construct (*SAL1-I* lines *1*, *28*, and *53*)*.* Finally, the cytosolic expression of *SAL1* at levels similar to those of the wild type (in lines *2* and *10*) (Figure 4B) was not sufficient to complement the biomass phenotype (Figure 5H) or the high PAP levels (Figure 5B) of *sal1*.

#### 2.3.3 Chloroplast

Surprisingly, the transformation of *sal1* by construct *SAL1_III* (targeting of *SAL1* to the chloroplast) only led to partial complementation across lines with different *SAL1* expression levels (Figure 4C). Multiple lines such as *SAL1_III 16*, *28*, *45*, and *53* showed full complementation of biomass, but not other parameters (Figures 3, 5C,I). Despite having comparable shoot fresh weight to that of wild-type plants (Figure 5I), the PAP levels in these lines were only partially decreased (Figure 5C) (from 17 to 5 pmol/mg) compared to those in *sal1*, and their rosette morphology was intermediate between that of Col-0 and *sal1*. As expected, two lines (*SAL1_III* line *26* and *SAL1_III* line *36*) with only moderately decreased PAP contents showed no complementation of biomass and rosette morphology (Figures 3, 5C,I).

#### 2.3.4 Mitochondria

Complementation of *sal1* by construct *SAL1_IV* (targeted expression of *SAL1* to the mitochondria) was extremely effective. Sixty-five out of 77 independent transgenic lines (85%) were fully complemented (data not shown). Here, we show six representative lines (Figures 4D, 5D,J) to illustrate the range of complementation observed. Lines *SAL1_IV 8*, *19*, *24*, and *26* had fully complemented PAP levels, whereas line *SAL1_IV 16* showed partial complementation and *SAL1_IV* line *15*, which had lower *SAL1* transcript levels than the wild type, was not complemented. The extent to which the PAP levels and rosette morphology were complemented correlated well with the PAP levels of these lines (Figures 3, 5D).

#### 2.3.5 Chloroplasts and Mitochondria

Complementation of *sal1* by construct *SAL1_V* (*SAL1* targeted to the mitochondria and chloroplasts) was functionally effective, as expected. Here, we present the data for nine representative independent transgenic lines with a range of phenotypes (Figures 4E, 5E,K). Six of these lines (*SAL1_V 1*, *13*, *15*, *18*, *24*, and *26*) showed wild-type phenotypes, including leaf blade shape (Figure 3), PAP levels (Figure 5E), and biomass (Figure 5K). Nevertheless, line *SAL1-V 6*, which had a comparable *SAL1* expression level to that of the wild type, was phenotypically similar to *sal1* in terms of biomass and rosette morphology, although the PAP level was moderately but significantly decreased. Only lines *SAL1_V 2* and *SAL1_V 17* were not complemented, presumably due to their very low *SAL1* expression (Figure 4E).

#### 2.3.6 Cytosol and Nucleus

Following transformation with *SAL1_VI,* which served as a positive control for complementation (Kim and von Arnim, 2009), we obtained 12 independent transgenic lines with a stable phenotype and analyzed nine representative lines in detail (Figures 3, 4F, 5F,L). Only one line (*SAL1_VI 6*) was complemented in terms of leaf morphology and growth (Figure 4) and shoot fresh weight (Figure 5L), with its PAP level almost restored to that of the wild type (Figure 5F). The remaining eight lines all had similar or significantly higher PAP levels compared to *sal1* and showed limited complementation in terms of rosette morphology and biomass (Figures 3, 4L). We did not expect to find only 1 out of 12 lines to be complemented or to show a wild-type phenotype because this construct was previously reported to be able to complement *sal1* (Kim and von Arnim, 2009). Thus, complementation of *sal1* by co-targeting SAL1 to the nucleus and the cytosol is possible in principle, but is not a guaranteed outcome (see Section 3).

### 2.4 The Sulfur Assimilation Pathway and Accumulation of Secondary Sulfated Compounds in Selected SAL1 Complemented Lines

**FIGURE 6:**
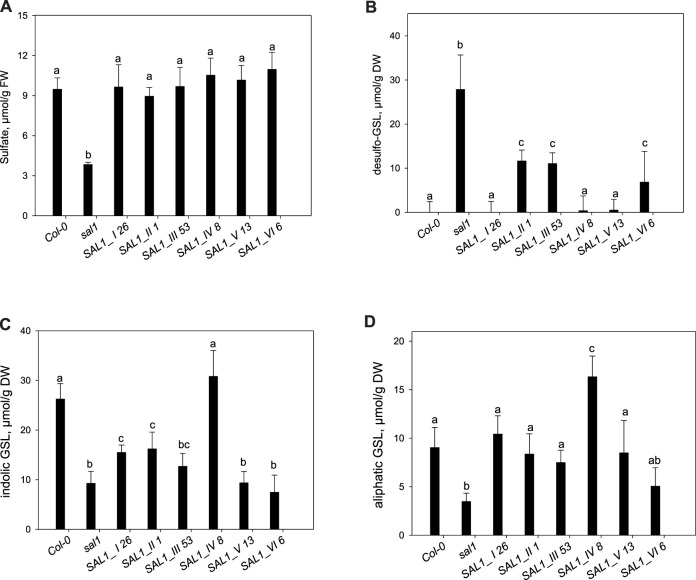
Analysis of sulfate, desulfo-glucosinolate (GSL), and aliphatic and indolic GSL levels in complemented *sal1* mutant lines. The levels of sulfate **(A)**, desulfo-GSL **(B)**, indolic GSL **(C)**, and aliphatic GSL **(D)** were analyzed in rosette leaves of 5-week-old *sal1* and complemented lines *SAL1_I 26*, *SAL1_II 1*, *SAL1_III 53*, *SAL1_IV 8*, *SAL1_V 13*, and *SAL1_VI 6.* Sulfur metabolites of partially complemented lines are shown in Supplementary Figure S3. Plants were grown on soil in short days in an environment-controlled chamber. Data show the mean ± SD (*n* = 4). *FW*, fresh weight. *Different letters* indicate significant differences among means based on *t*-tests at *p* < 0.05.

Loss of SAL1 function results in low total sulfate levels, a decreased accumulation of GSLs, an increased level of desulfo-precursors, and a decreased level of thiols (Lee et al., 2012). Furthermore, sulfur assimilation in *sal1* mutants was not only affected at the metabolic level, but the transcript profile of genes was similar to that of sulfate-starved plants (Lee et al., 2012). Therefore, in addition to the general processes related to growth and stress response, the nutritional status was also impaired in *sal1* plants. To address the role of the compartmentalization of SAL1 in sulfur assimilation, we selected the best-complemented transgenic lines, *SAL1_I 26*, *SAL1_II 1*, *SAL1_III 53*, *SAL1_IV 8*, *SAL1_V 13*, and *SAL1_VI 6* (Figures 4, 5), and analyzed their sulfur metabolite profiles (Figure 6). The accumulation of sulfur metabolites in phenotypically weakly complemented lines is shown in Supplementary Figure S3.

The sulfate content in the selected lines revealed that *SAL1* can rescue the low-sulfate phenotype of *sal1* in all compartments, even when it is expressed in the cytosol or the nucleus alone (Figure 6A). However, in weakly expressing lines, complementation of low sulfate levels only occurred when *SAL1* was expressed in the nucleus, mitochondria, or chloroplasts, but not when SAL1 was present in the cytosol (constructs II and VI) (Supplementary Figure S3).

As sulfate is required for the sulfation of GSLs, we additionally measured the accumulation of indolic (IG) and aliphatic (AG) GSLs and their desulfo-precursors. The desulfo-GSLs (ds-GSLs), which accumulate to high levels in *sal1*, were fully complemented by *SAL1* expression in the nucleus, mitochondria, and by the native *SAL1* construct. However, the cytosolic and chloroplastic expression of *SAL1* only led to a 50% decrease in the accumulation of ds-GSL, which could not restore the wild-type level of ds-GSL (Figure 6B). In contrast to ds-GSLs, we observed less phenotypic variation among the SAL1-expressing lines for AGs and IGs. The production of AGs was fully complemented in all lines, with the exception of line VI in which the low AG level only slightly increased (Figure 6D). Conversely, the production of IGs was fully complemented only when SAL1 was targeted to the mitochondria (Figure 6C).

### 2.5 Phenotypic Comparison of Complementation Efficiency by SAL1 in Different Subcellular Compartments

**FIGURE 7:**
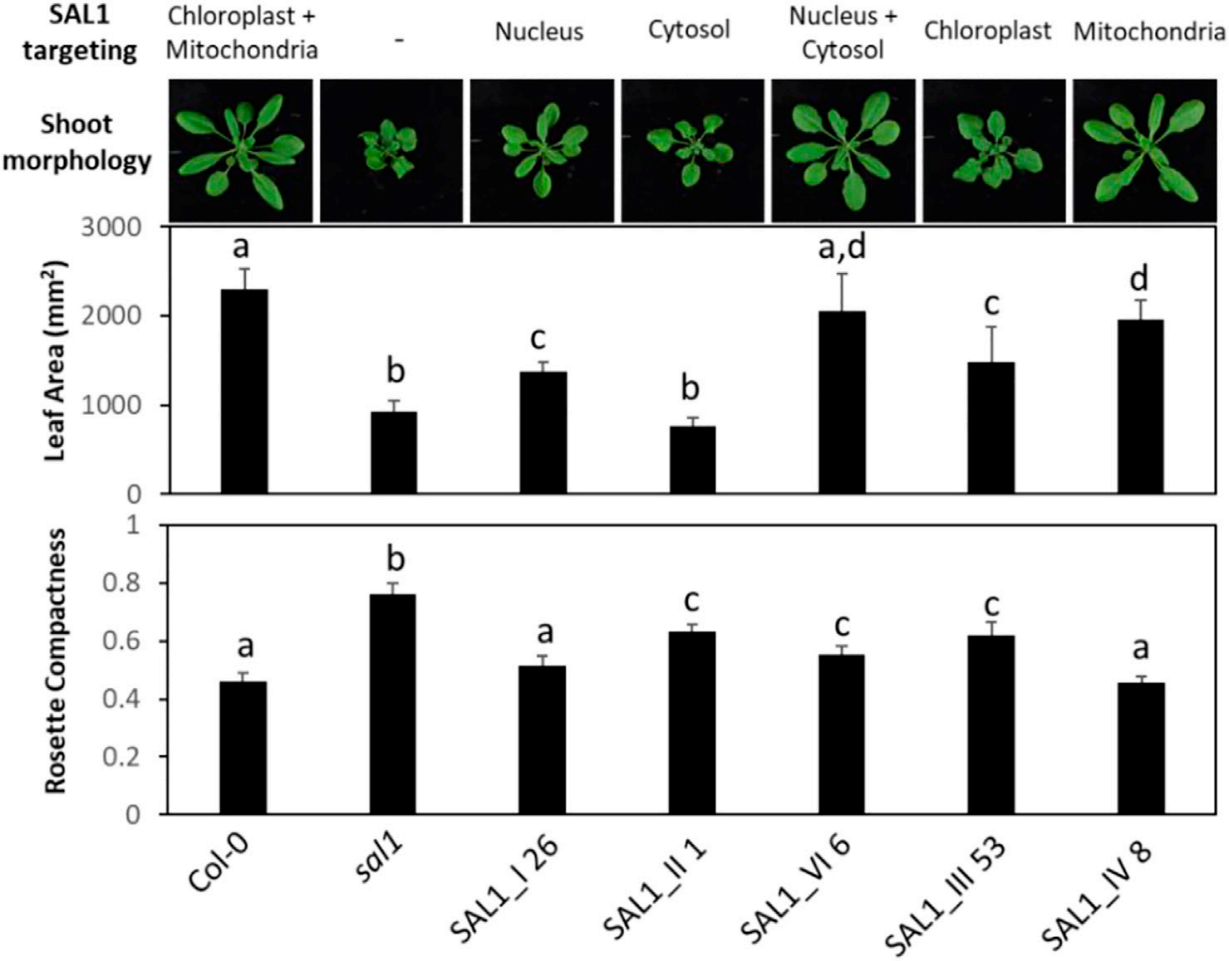
Shoot morphology, leaf area, and rosette compactness in best-complemented *sal1* mutant lines expressing SAL1 in different subcellular compartments. Morphological phenotypes were analyzed in rosette leaves of 25-day-old soil-grown plants. The entire aboveground rosette was excised and photographed under standardized lighting conditions, then the image analyzed using ImageJ to quantify the leaf area and rosette compactness. Data show the mean ± SD (*n* = 5). *Different letters* indicate significant differences among means based on one-way ANOVA and Tukey’s honestly significant difference (HSD) *post-hoc* test at *p* < 0.05. Note that line V_13 was not analyzed here due to the impaired germination and growth of the seeds after prolonged storage between the initial characterization (Figures 2–6) and this analysis. Furthermore, given that V_13 utilizes the native SAL1 targeting sequence (Figure 1), in our view, V_13 should be functionally similar to the wild-type control. Thus, the wild type and constructs III and IV are sufficient for comparing SAL1 targeting to the chloroplasts, mitochondria, or both organelles.

We further characterized the best-complemented lines in Figure 6 by quantifying their leaf phenotypes in more detail. We did comparative analysis of the following transgenic lines: 1) plants expressing SAL1 in the compartments in which PAP is proposed to act (nucleus, cytosol, or both) compared to the wild type and 2) plants expressing SAL1 in a single organelle (chloroplast or the mitochondria) compared to both organelles where SAL1 is normally found (wild type). Targeting SAL1 to both the cytosol and the nucleus (*SAL1_VI_6*) showed an additive effect compared to either the nucleus (*SAL1_I 26*) or the cytosol (*SAL1_II_1*) alone in restoring leaf area to wild-type levels (Figures 6, 7). In contrast, SAL1 in either the cytosol or the nucleus alone was similarly effective as SAL1 in both the cytosol and the nucleus in rescuing rosette compactness (Figure 7). In the second set of comparisons, targeting SAL1 to chloroplasts alone (*SAL1*_*III 53*) only partially rescued leaf area and rosette compactness. Similarly, targeting SAL1 to the mitochondria (*SAL1_IV*) did not completely restore leaf area, although it rescued rosette compactness to wild-type levels (Figure 7). Taken together, these results suggest that PAP most likely exerts its effects in both the cytosol and the nucleus, albeit unequally, and that the presence of SAL1 in both chloroplasts and the mitochondria is required for PAP homeostasis.

### 2.6 Re-Evaluating Complementation Efficiency by SAL1 in the Context of *SAL1* mRNA and Protein Levels *In Vivo*

The unequal complementation obtained with the different constructs (Figures 3–7) was striking and unexpected, given that *SAL1* was driven by a strong promoter that, in theory, should drive overexpression in all constructs. Therefore, we first examined for any possible relationship between the different phenotypes and *SAL1* mRNA levels in lines of the different constructs. *SAL1* overexpression at 5- to 10-fold of the wild-type levels was sufficient to completely restore the PAP levels in *SAL1_IV 26*, but not in lines of the other constructs such as *SAL1_I 53*, *SAL1_II 2*, *SAL1_III 53*, and *SAL1_VI 5* (Figures 4, 5). Similarly, *SAL1* expression at levels comparable to those of wild-type Col-0 restored plant biomass and significantly decreased the PAP levels only in construct *IV*, but not in lines of the other constructs (Figures 4, 5).

**FIGURE 8:**
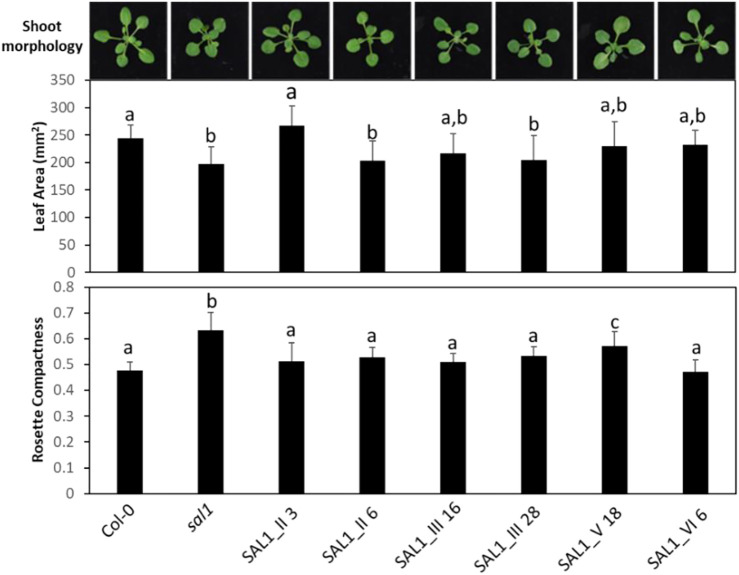
Shoot morphology, leaf area, and rosette compactness in complemented *sal1* mutant lines expressing *SAL1* mRNA at similar levels. Morphological phenotypes were analyzed in rosette leaves of 25-day-old soil-grown plants. The entire aboveground rosette was excised and photographed under standardized lighting conditions, then the image analyzed using ImageJ to quantify the leaf area and rosette compactness. All lines shown here have *SAL1* mRNA overexpressed at ∼10-fold the levels of Col-0. Data show the mean ± SD (*n* = 5). *Different letters* indicate significant differences among means based on one-way ANOVA and Tukey’s honestly significant difference (HSD) *post-hoc* test at *p* < 0.05. Note that, similar to Figure 7, there was either complete or serious loss in germination rate and/or seedling growth in lines of interest, which were not seen in earlier experiments (e.g., lines I_61, IV_19, IV_26, and V_13). We decided to omit representatives from certain lines (e.g., construct IV) because no other representative within that construct had similar SAL1 mRNA expression to the rest of the constructs. However, we attempted to compensate for this deficiency by studying two representatives of certain constructs (II and III), in cases where they have similar SAL1 mRNA expression to the rest of the constructs.

**FIGURE 9:**
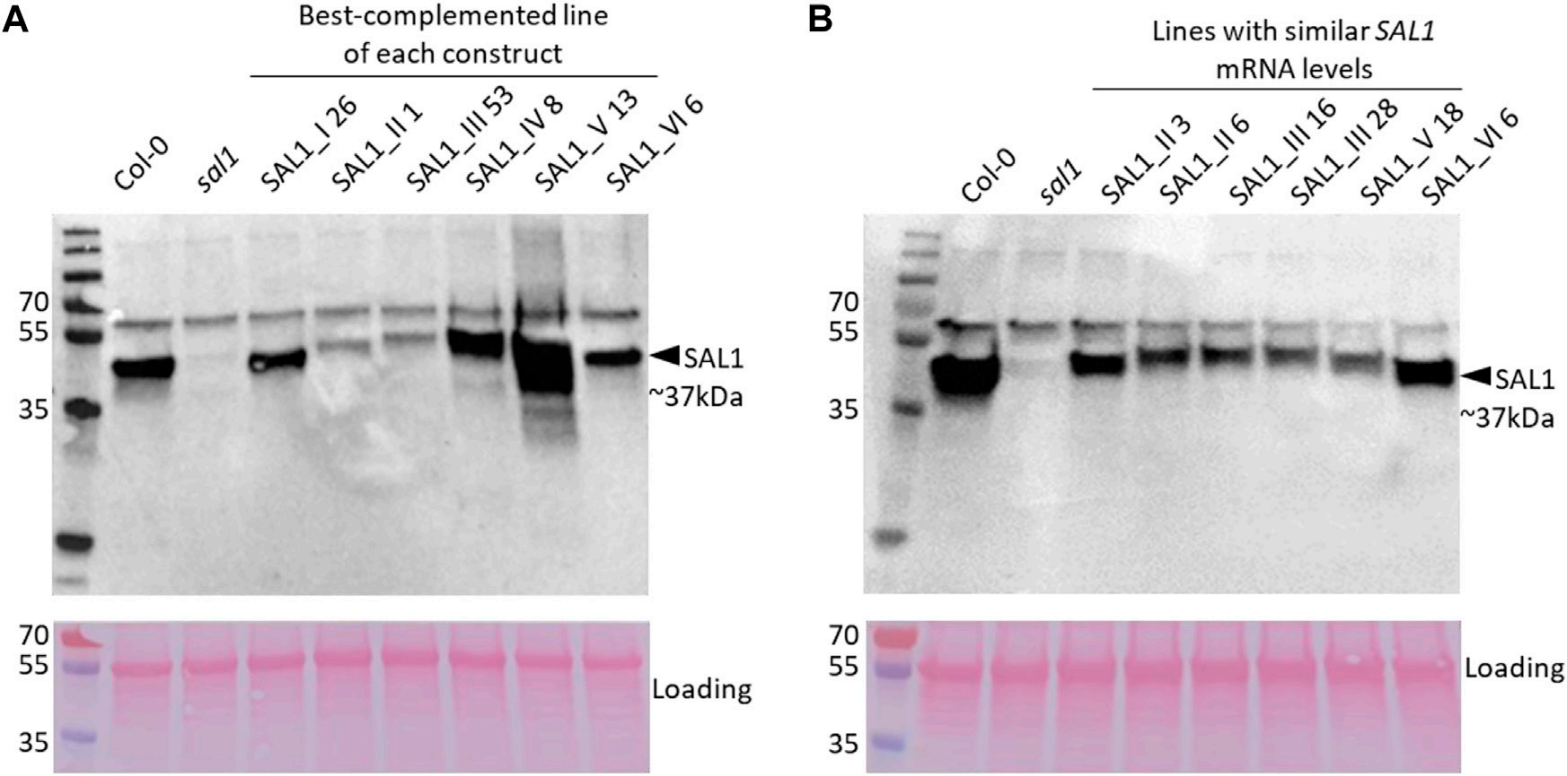
SAL1 protein levels in best-complemented *sal1* lines expressing SAL1 in different subcellular compartments and in complemented *sal1* lines expressing similar *SAL1* mRNA levels. SAL1 protein levels were analyzed in total protein extracts of 25-day-old rosette leaves from the best-complemented line of *sal1* expressing the different constructs I–VI **(A)** and diverse complemented *sal1* lines with similar *SAL1* mRNA levels (∼10-fold overexpression compared to Col-0) **(B)**. Immunoblotting was performed with an anti-SAL1 antibody and a horseradish peroxidase (HRP)-conjugated secondary antibody against 20 µg total protein per sample. The SAL1 protein was detected at approximately 37 kDa, with no SAL1 protein in the *sal1* mutant sample, as expected. A higher band at 55 kDa is most likely the result of nonspecific antibody binding to Rubisco (*upper panels*). The blots were subsequently stained with Ponceau S and re-imaged to evaluate equal loading between samples (*lower panels*). In both **(A)** and **(B)**, the *upper* and *lower* panels were generated from the same blot that was independently imaged twice after incubation with HRP chemiluminescence substrate and Ponceau S, respectively.

To address the possibility that the mRNA levels of transgenic *SAL1* are uncoupled from the protein levels of SAL1 in the different constructs, we first compared selected lines with similar *SAL1* mRNA expression levels (∼10-fold higher than that of Col-0) irrespective of their targeting. The different constructs showed significantly different degrees of leaf complementation despite their similar levels of *SAL1* mRNA (Figure 8). Interestingly, the variance in leaf phenotype complementation appeared linked to substantial variation in the protein levels of SAL1, with the Western blot of lines *SAL1_III 16* and *28* in particular showing much weaker SAL1 protein bands compared to *SAL1_II 3* and *SAL1_VI 6* (Figure 9B). This was further confirmed by comparing the protein and mRNA levels of SAL1 in the best-complemented lines of each construct regardless of their *SAL1* mRNA levels (Figure 9A). Although the *SAL1_I 26*, *III 53*, and *VI 6* lines all expressed *SAL1* mRNA at 10- to 15-fold that of the wild type, their SAL1 protein levels varied substantially, with *III 53* showing very low SAL1 protein abundance. *SAL1_II 1* had similar SAL1 protein abundance to *III 53* despite overexpressing *SAL1* mRNA to a greater extent (40- *vs*. 10-fold, respectively). Similarly, *SAL1_V 13* accumulated substantially more SAL1 protein than *SAL1 IV 8* despite overexpressing *SAL1* mRNA to a lesser degree (60 *vs*. 80-fold). Therefore, instead of *SAL1* mRNA being the primary determinant of complementation, targeting SAL1 to different subcellular compartments may lead to different levels of the SAL1 protein *in vivo* through unknown mechanism(s), thus causing unequal complementation.

## 3 Discussion

The accumulation of PAP differs in different subcellular compartments due to the activities of SAL1. Based on current knowledge concerning the SAL1–PAP pathway and taking all phenotypes reported for *sal1* mutants into consideration, we hypothesized that PAP can act in different compartments, where PAP signaling can potentially execute different functions (Phua et al., 2018). These intriguing scenarios prompted us to design experiments addressing the role of SAL1 in different cell compartments by expressing it in chloroplasts, the mitochondria, the nucleus, and the cytosol. This set of experiments aimed to compare the ability of *SAL1* expressed in different compartments to complement the morphological, physiological, and chemical phenotypes of *sal1*, thereby revealing initial hints on how PAP might differentially affect multiple growth- and sulfur metabolism-related phenotypes.

Our results indicated that SAL1 expression in the nucleus (construct I) was sufficient to complement the *sal1* phenotypes (Figures 4–7). This finding, which is consistent with previous observations by Kim and von Arnim (2009), pointed that: 1) PAP is present in the nucleus due to diffusion from the cytosol and that 2) modulating nuclear the PAP levels can revert the phenotype of *sal1* to that of the wild type. This finding is also consistent with the localization of two known PAP targets, XRN2 and XRN3, in the nucleus. PAP inhibits the 5′–3′ exoribonuclease activity of the XRN proteins, most likely by binding to the active site of XRNs (Nagarajan et al., 2013), as suggested by *in vitro* assays and the protein crystallography of PAP-inhibited exoribonucleases (Yun et al., 2018). In contrast, the high mRNA level of cytosol-targeted SAL1 did not fully complement *sal1* with respect to leaf shape, PAP level, plant biomass, and GLs (*SAL1_II* lines; Figures 4–6). This is counterintuitive because PAP is generated in the cytosol and, therefore, the cytosolic expression of SAL1 was predicted to directly detoxify PAP without the requirement for PAP transport into organelles or its diffusion into the nucleus. Similarly, targeting SAL1 to the chloroplast, which is one of the organelles in which SAL1 is normally located in wild-type plants, should have enabled efficient complementation of *sal1* phenotypes. These discrepancies can be explained by the low levels of transgenic SAL1 protein when expressed exclusively in the cytosol or in the chloroplast (Figure 9). It is also possible that the introduction of a NES or the Rubisco SSU targeting peptide to the SAL1 sequences in *SAL1_II* and *SAL1_III* lines, respectively, could have negatively impacted its protein folding or structure since even relatively few (one to four) codon changes to the SAL1 sequence are sufficient to decrease its protein abundance in *Arabidopsis* (Wilson et al., 2009) and *Escherichia coli* (Chan et al., 2016). Interestingly, in an experiment aiming at the subcellular localization of native SAL1_V_GFP, we also observed low levels of the GFP protein, which can be caused by both GFP and NES. Although the NES was also present in SAL1 sequences of the well-complemented *SAL1_IV 8* and *SAL1_V 13* lines, these two lines had among the highest levels of *SAL1* mRNA overexpression across all the lines tested, which could have partially compensated for the decreased protein stability.

As demonstrated by line *SAL1_IV 8*, all secondary sulfate metabolism metabolites, including sulfate, ds-GSL, and aliphatic and indolic GSLs, were also fully restored to wild-type levels (Figure 6). Notably, sulfur assimilation (Supplementary Figure S3) was highly stimulated by the mitochondrial expression of SAL1. Aliphatic and indolic GSLs reached levels significantly higher than those in the wild type (Figures 6C, D). Taken together, these observations confirmed the transport of PAP into the mitochondria for degradation by SAL1, which was previously only inferred based on the localization data for SAL1 and the PAPS/PAP transporter PAPST2 (Estavillo et al., 2011; Ashykhmina et al., 2019). Nevertheless, PAP degradation by SAL1 in both chloroplasts and the mitochondria may still be required for complete regulation of PAP-mediated signaling since the leaf area was still significantly, albeit only slightly, lower in *SAL1_IV 8* compared to the wild type (Figure 9).

When comparing the PAP levels, leaf area, and rosette compactness between the different lines (Figures 5, 7), it appears that the same level of PAP may exert different impacts on individual growth phenotypes. A moderately elevated PAP level compared to that of the wild-type was sufficient to suppress the altered leaf area, but not rosette compactness (e.g., *SAL1_V 13* and *SAL1_VI 6*), whereas higher PAP levels that were intermediate between the wild type and *sal1* also had an impact on rosette morphology (*SAL1_II 1* and *SAL1_III 53*). Similarly, the lines *SAL1_II 6*, *SAL1_III 16*, and *SAL1_III 28*, all of which have similar PAP contents, have suppressed leaf area like *sal1*, but have wild type-like rosette compactness. Notably, rosette compactness is highly influenced by petiole length (Vanhaeren et al., 2015), which is regulated by light signaling components such as PhyB that are also influenced by PAP accumulation (Kim and von Arnim, 2009). Leaf area is a function of cell size and number, which in turn are regulated by several complex factors such as cell expansion, cell wall composition, and the cell cycle (Gonzalez et al., 2012). How PAP signaling influences these processes is still not clear. Given that a small increase in cytosolic PAP levels, as observed here for the *SAL1_IV* lines or in the *papst2* mutant (Ashykhmina et al., 2019), actually enhances plant growth while further PAP elevations suppress growth, it may be that at least one of the parameters governing cell growth and replication is highly tuned to fluctuations in the intracellular PAP levels.

Similarly, the production of indolic GSLs seemed more sensitive to PAP accumulation than that of aliphatic GSLs. Both *SAL1_II 1* and *SAL1_III 53* had only partially complemented ds-GSLs and indolic GSLs, yet exhibited complete complementation of aliphatic GSLs. Given that SOT16 highly prefers indolic ds-GSLs whereas SOT18 prefers aliphatic ds-GSLs *in vitro* (Klein and Papenbrock, 2009), it is possible that PAP exerted a stronger inhibitory effect on SOT16 than on SOT18 *in vivo*. The inhibition kinetics of PAP have only been determined for SOT18 so far (Hirschmann et al., 2017). Alternatively, it may be that the effect of PAP on indolic GSLs was more pronounced due to their greater abundance *in vivo* compared to aliphatic GSLs (Figure 6).

All of the best-complemented lines from each construct showed complete recovery of the sulfate levels. This suggests that the regulation of total sulfate is relatively insensitive to PAP accumulation until a very high threshold is reached, as shown by *SAL1_II 1*, which had wild-type sulfate content despite accumulating 50% of the PAP levels in *sal1* (Figures 5, 6). Recently, it has been shown that GSLs serve as a sulfate reservoir and can be catabolized to release free sulfate (Sugiyama et al., 2021), so it is tempting to speculate that a high PAP may affect the sulfate levels at least partly *via* GSLs. It is also possible that PAP affects sulfate by modulating sulfate transport since the expressions of some sulfate transporters were affected in *fry1-6* (Estavillo et al., 2011).

Collectively, our results indicate that the role of the SAL1–PAP pathway in different cell compartments, environmental conditions, and signaling pathways is likely to be more complex than previously assumed. Understanding how PAP exerts differential effects on diverse processes, such as leaf morphology, growth, and sulfur metabolism, as we have shown here, could be the key to unraveling its role in the integration of retrograde, light, and hormonal signaling (Rodríguez et al., 2010; Estavillo et al., 2011; Ishiga et al., 2017; Phua et al., 2018), as well as in nutrient (Hirsch et al., 2011; Lee et al., 2012) and pathogen signaling (Ishiga et al., 2017).

## 4 Materials and Methods

### 4.1 Plant Material and Growth Conditions

Seeds of *A. thaliana* ecotype Col-*0*, the T-DNA insertion mutant, and complemented transgenic plants were sown on soil or culture plates containing 1/2 Murashige and Skoog medium. The seeds were stratified for 2–3 days in the dark, and the plants were cultivated under short-day (8-h light and 16-h dark) conditions with an average photon flux density of 100–150 μmol m^−2^ s^−1^. White light was provided by Fluora L58W/77 fluorescent tubes. The temperature was kept at 22°C during the light period and 18°C during the dark period. The relative humidity was ∼40%.

### 4.2 Isolation of *sal1* Mutant

The homozygous mutant *sal1* line (At5g63980, SALK_020882, and SALK_005741) was identified and the insertion position of the T-DNA in the target gene was confirmed by sequencing previously (Ashykhmina et al., 2019). In this manuscript, we present data on the analysis and complementation of SALK_020882 line, named here also as *sal1*.

### 4.3 Cloning of Chimeric *SAL1* Constructs

To achieve the compartment-specific accumulation of *SAL1*, we generated six chimeric constructs, as shown in Supplementary Figure S1. The *SAL1tr* backbone was amplified from *Arabidopsis* cDNA and fused with different DNA pieces using fusion PCR, as described in *Section 2*.

In our cloning procedure, all six constructs were inserted into the entry pDONOR207 vector. pDONOR207 vectors containing six different SAL1s were recombined with either the binary vector pGWB5 (for GFP localization studies) or pGWB2 (for the complementation of *sal1*) under the control of the 35S cauliflower mosaic virus promoter. All constructs of interest were transformed into *Arabidopsis* mesophyll protoplasts or root suspension cells.

### 4.4 GFP-Based Subcellular Localization of *SAL1* Proteins in *Arabidopsis* Protoplasts and in *Arabidopsis* Suspension Cell Culture From Roots

To confirm the subcellular localization of *SAL1* fusion proteins experimentally, *Arabidopsis* mesophyll protoplasts and root cell suspension cultures were transformed with *Agrobacterium* carrying constructs encoding SAL1_I–VI fused to the GFP at the C-terminus. Transfection of *Arabidopsis* mesophyll protoplasts was performed as described by Yoo et al. (2007) using 20–40 μg of plasmid DNA. Transformation of *Arabidopsis* root suspension cells was performed as described previously (Berger et al., 2007).

For staining with 4′,6-diamidino-2-phenylindol (DAPI), *Arabidopsis* cells were incubated in 2 μg ml^−1^ (*w*/*v*) solution of DAPI for 5 min in the dark, followed by one to two times rinsing with cell culture media (Berger et al., 2007). For MitoTracker staining, the cells were removed from the medium and exposed in 500 nmol concentrated MitoTracker dye for 45–60 min. Cells were rinsed several times with cell culture media prior to imaging.

The GFP expression patterns in dark-grown cultured *Arabidopsis* or protoplasts from mesophyll tissue were recorded by confocal laser scanning microscopy (Zeiss, Jena, Germany). Images were acquired as *z*-series with a 1- to 3-μm interval with 25 frames using a Zeiss LSM510 confocal laser scanning microscope. Green fluorescent protein was visualized with a 488-nm laser and a band-pass (BP) 500–530 filter and the MitoTracker stain with a 543-nm laser and a BP 565–615 filter. For the visualization of DAPI, we used ultraviolet light and a BP 385–470 filter.

Co-localization of the GFP signal with chlorophyll autofluorescence indicated chloroplastidic localization. A co-labeling of GFP with the DNA fluorescent stain DAPI was interpreted as a nuclear localization, whereas a co-labeling of GFP with the MitoTracker indicated a mitochondrial localization.

Results were documented with Discus and Zeiss software. Images were processed using Photoshop CS3 (Adobe Systems, San Jose, CA, USA). Adjustment of levels was applied to the SAL1-V construct to make weak GFP signals better visible in protoplasts.

### 4.5 Complementation of *sal1* Mutant Using *SAL1 I* to *VI* Constructs

To complement *sal1*, we utilized constructs *SAL1 I* to *VI* without GFP and generated stable *Arabidopsis* transgenic lines. All six SAL1 constructs were recombined from pDONR207 into the Gateway destination pGWB2 vector (35S cauliflower mosaic virus promoter), and correctness of constructs was verified by sequencing. *SAL1 I* to *VI* were delivered to *sal1 Arabidopsis* plants by *Agrobacterium*-mediated transformation, and positive transformants were selected using kanamycin. The homozygous *SAL1ox* lines *I* to *VI* were isolated following segregation analysis of populations.

### 4.6 Analysis of Shoot Fresh Weight of Complemented Plants

Individual *Arabidopsis* plants were grown on MS agar media for 4 weeks as described above. To measure the shoot fresh weight of complemented *sal1* mutants, the aboveground rosettes were excised at the hypocotyl and individual weight was recorded. At least nine seedlings per construct were analyzed.

### 4.7 RNA Extraction and RT-qPCR

To measure the transcript levels in the wild type and the different complemented mutant plants, total RNA was isolated from leaves, cDNA was reversely transcribed, and reverse transcription quantitative PCR (RT-qPCR) was performed as described previously (Gigolashvili et al., 2009). The relative quantification of the expression levels was performed using the comparative delta *C*_t_ method, and the calculated relative expression values were normalized to *Actin2* and compared with the expression level in wild-type plants (Col-0 = 1).

### 4.8 Extraction and Measurement of Sulfated Compounds and of Sulfate

GSLs and their desulfo-precursors were extracted from the lyophilized plant material as reported previously (Ashykhmina et al., 2019). Sulfate was detected as previously reported in Mugford et al. (2009).

### 4.9 Quantification of PAP in Plant Extracts

Plant material was collected and frozen in liquid nitrogen. The extraction and chromatographic detection of PAP were performed as reported previously (Ashykhmina et al., 2019).

### 4.10 Quantification of Rosette Parameters

Individual *Arabidopsis* plants were grown on separate soil-filled pots for 25 days under long-day conditions (18-h light and 6-h darkness at ∼100 µmol photons m^−2^ s^−1^, 22°C/20°C day/night cycle). For imaging, the aboveground rosettes were excised at the hypocotyl with sharp scissors and immediately photographed using an in-house imaging platform comprising a Canon DSLR camera and controlled fluorescent lighting. The images were analyzed in ImageJ to quantify the leaf area (area of green pixels) and the rosette compactness (ratio of leaf area relative to the convex hull) (Vanhaeren et al., 2015).

### 4.11 Protein Extraction and Western Blot

Approximately 100 mg leaf tissue harvested from 25-day-old soil-grown *Arabidopsis* plants was snap-frozen in liquid nitrogen, ground to fine powder using a ball mill at 20 Hz for 1 min (Retsch, Haan, Germany), and then resuspended in 300 µl RIPA extraction buffer (1% NP-40, 0.5% sodium deoxycholate, 0.1% sodium dodecyl sulfate, and 10% glycerol) supplemented with 1 mM phenylmethylsulfonyl fluoride (PMSF). Cellular debris was removed by centrifugation (14,000 × g for 10 min at 4°C) and the supernatant moved to a fresh tube. Total protein was quantified using the Bradford assay. For SDS-PAGE and Western blotting, 20 µg total protein was loaded per sample onto 12% Mini-PROTEAN® TGX™ Precast Gels (Bio-Rad, Hercules, CA, United States) and resolved at 200 V for 30 min. The proteins were then transferred unto PVDF membranes using the Trans-Blot Turbo Transfer System (Bio-Rad). The membranes were incubated in blocking solution (5% milk, 1 × PBS, and 0.05% Tween) for 1 h at room temperature. This was followed by an anti-SAL1 antibody (Agrisera AS07 256; 1:1,000 dilution in 1% milk, 1 × PBS, and 0.05% Tween) overnight at 4°C and a secondary antibody (ECL^TM^ Donkey anti-rabbit IgG, horseradish peroxidase linked; GE Healthcare, Chicago, IL, United States) for 1 h at room temperature, with three 15-min washes (1 × PBS and 0.05% Tween) after every incubation step. The blots were developed using ECL imaging solution (Clarity Western Peroxide Reagent and Clarity Western Luminol/Enhancer Reagent; Bio-Rad). Chemiluminescence was visualized using the ChemiDoc system (Bio-Rad). To visualize all proteins present on the blot, the membrane was incubated for 5 min in Ponceau S solution (Sigma-Aldrich, St. Louis, MO, USA) and washed with distilled water to remove background staining.

### 4.12 Statistical Analysis

Comparison of means was performed to determine statistical significance using a two-sample Student’s *t*-test or an ANOVA (Tukey’s test). Constant variance and normal distribution of data were verified prior to statistical analysis.

In the first phase of the project, comparison to wild type with repeated *t*-tests seemed to be the most accessible method of choice, as we were most interested in whether an individual line was not significantly different from the wild type (indicating complete complementation) rather than possible differences between certain lines (partial complementation). Prior to the *t*-test, a two-sample *F*-test for variances was implemented to check for scedasticity, shown in Figures 4–6. If the variances differed, a heteroscedastic two-tailed *t*-test was performed, while equal variances led to the choice of a homoscedastic *t*-test. For Figures 7, 8, homogeneity of the variances was verified using the Levene’s test for equality of variances in SPSS Statistics version 27 (IBM, Armonk, NY, USA). All datasets passed the test for equal variances, returning significance values between 0.3 and 0.6 (significance values higher than 0.05 indicate equal variance). The datasets were subsequently analysed by ANOVA.

## Data Availability

The original contributions presented in the study are included in the article/Supplementary Material. Further inquiries can be directed to the corresponding author.
