# Supplementary material for: Dissecting the Role of SAL1 in Metabolizing the Stress Signaling Molecule 3′-Phosphoadenosine 5′-Phosphate in Different Cell Compartments
Source: Front Mol Biosci. 2022 Jan 21;8:763795. doi: 10.3389/fmolb.2021.763795 (PMC8815814; doi:10.3389/fmolb.2021.763795)
Supplement: Supplementary file 1 [file DataSheet1.docx]

**Supplemental Figures**


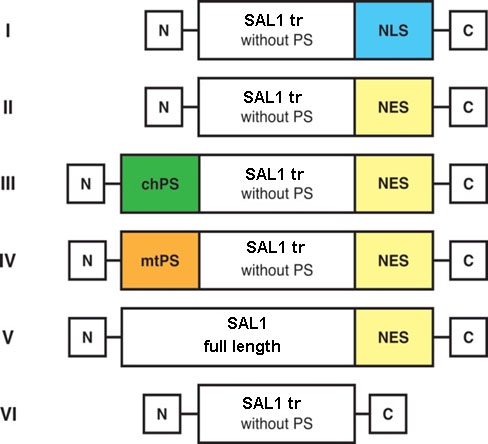


**Supplemental Figure S1. Schematic representation of SAL1 constructs**

Constructs were generated to target SAL1 protein to different cell compartments. **I**. Nuclear localization; truncated SAL1 (SAL1tr) with the C-terminal nuclear localization sequence (NLS). **II.** Cytosolic localization; SAL1tr fused to C-terminal Nuclear Exclusion Sequence (NES). **III.** Chloroplastic localization; SAL1tr with N-terminal chloroplast pre-sequence (cPS) and C-terminal NES. **IV**. Mitochondrial localization; SAL1tr with N-terminal mitochondrial PS (mPS) fused to C-terminal NES. **V.** Chloroplastic and mitochondrial localization; full-length SAL1 (SAL1fl) fused to C-terminal NES. **VI**. Nuclear and cytosolic localization as described by ([Kim and von Arnim, 2009](#_ENREF_15)); SAL1tr. chPS – chloroplastidic localization (pre)sequence; mtPS – mitochondrial localization (pre)sequence; PS- (pre)sequence.


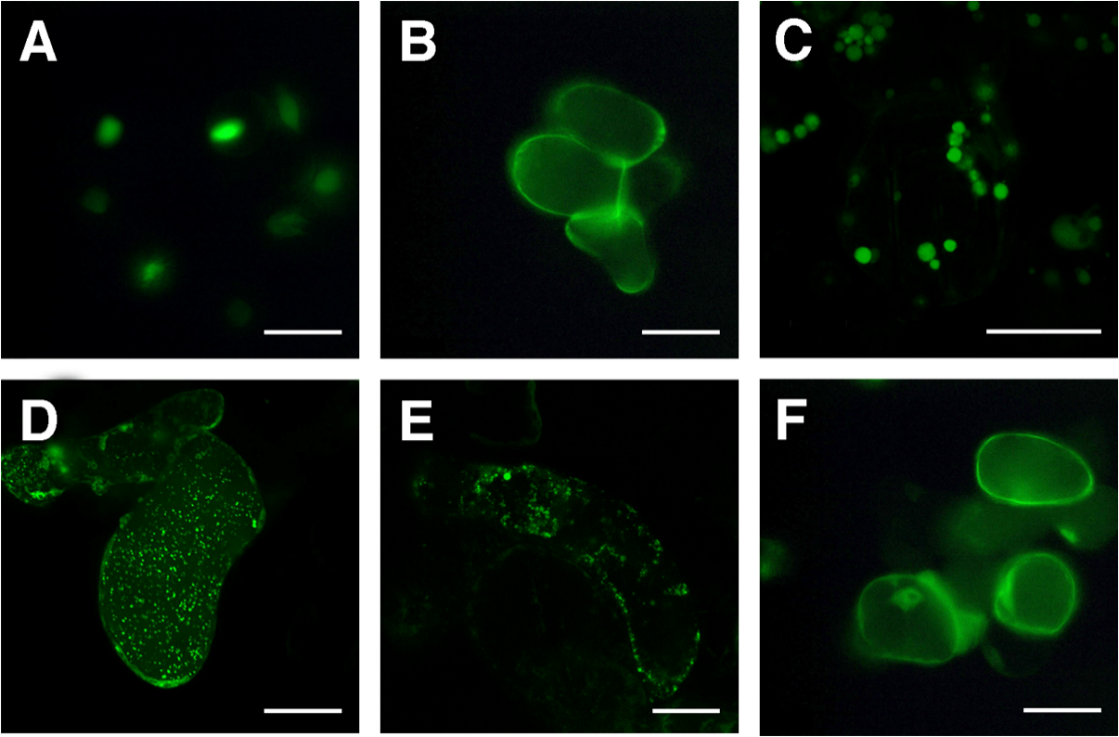


**Supplemental Figure S2.** **Subcellular localization of *SAL1_I* - *SAL1_VI* proteins in Arabidopsis root cell suspension cultures**

Transient expression of SAL1 protein chimeras fused to GFP under control of the *35S CaMV* promoter in various sub-cellular compartments of cells from *Arabidopsis* root-cell suspension culture as revealed by confocal fluorescence microscopy: **(A)** SAL1_I:GFP, **(B)** SAL1_II:GFP, **(C)** SAL1_III:GFP, **(D)** SAL1_IV:GFP, **(E)** SAL1_V:GFP, **(F)** SAL1_VI:GFP. Bar = 20 μm.

| **A**   | **B**   |
| --- | --- |
| **C**   | **D**   |

**Supplemental Figure S3. Levels of sulfate, desulfo-GSL, aliphatic GSL and indolic GSL in *sall1* mutants expressing *SAL1* in various cell compartments**

Levels of sulfate **(A)**, desulfo-GSL **(B)** aliphatic GSL **(C)** and indolic GSL **(D)** in rosette leaves of 5-week-old *sal1* mutants and stably transformed *sal1* transgenic plants overexpressing SAL1 in various cell compartments. *SAL1_I* – nuclear localization; *SAL1_II* – cytosolic localization; *SAL1_III* – chloroplastic localization; *SAL1_IV* – mitochondrial localization; *SAL1_V* – chloroplastic and mitochondrial localization; *SAL1_VI* – nuclear and cytosolic localization. Two lines for each compartment were analyzed, having low or high relative *SAL1* level. Plants were grown in soil in short days in a controlled environment chamber. Data show means ± SD (n = 3). DW = dry weight. Different letters indicate significant differences among means based on *t*-tests at *P* < 0.05
